# The Effects of Animacy and Syntax on Priming: A Developmental Study

**DOI:** 10.3389/fpsyg.2017.02246

**Published:** 2017-12-20

**Authors:** Leone Buckle, Elena Lieven, Anna L. Theakston

**Affiliations:** ESRC International Centre for Language and Communicative Development (LuCiD), School of Health Sciences, University of Manchester, Manchester, United Kingdom

**Keywords:** structural priming, animacy, language production, language acquisition, semantic roles, syntax

## Abstract

Sentence production relies on the activation of semantic information (e.g., noun animacy) and syntactic frames that specify an order for grammatical functions (e.g., subject before object). However, it is unclear whether these semantic and syntactic processes interact and if this might change over development. We thus examined the extent to which animacy-semantic role mappings in dative prime sentences and target scenes influences choice of syntactic structure (structural priming, analysis 1) and ordering of nouns as a function of animacy (animacy noun priming, analysis 2) in children and adults. One hundred forty-three participants (47 three year olds, 48 five year olds and 48 adults) alternated with the experimenter in describing animations. Animacy mappings for themes and goals were either prototypical or non-prototypical and either matched or mismatched across the experimenter's prime scenes and participants' target elicitation scenes. Prime sentences were either double-object datives (DOD e.g., *the girl brought the monkey a ball*) or prepositional datives (PD e.g., *the girl brought the ball to the monkey*), and occurred with either animate-inanimate or inanimate-animate, post-verbal noun order. Participants' target sentences were coded for syntactic form, and animacy noun order. All age groups showed a structural priming effect. A significant interaction between prime structure, prime animacy-semantic role mappings and prime-target match indicated that animacy could moderate structural priming in 3 year olds. However, animacy had no effect on structural priming in any other instance. Nevertheless, production of DOD structures was influenced by whether animacy-semantic role mappings in primes and target scenes matched or mismatched. We provide new evidence of animacy noun order priming effects in 3 and 5 year olds where there was prime-target match in animacy-semantic role mappings. Neither prime animacy noun ordering nor animacy-semantic role mappings influenced adults' target sentences. Our results demonstrate that animacy cues can affect speakers' word order independently of syntactic structure and also through interactions with syntax, although these processes are subject to developmental changes. We therefore, suggest that theories of structural priming, sentence production, linguistic representation and language acquisition all need to explicitly account for developmental changes in the role of semantic and syntactic information in sentence processing.

## Introduction

In order to communicate ideas, speakers must map concepts to syntactic structures. Where one idea can be expressed using multiple structures, speakers have a tendency to use the most recently heard structure (Bock, 1986). This is the structural priming effect. For example, the transfer of a ball between a girl and a monkey may be described with the double-object dative (DOD) sentence *the girl brought the monkey a ball* or the prepositional dative (PD) structure *the girl brought a ball to the monkey*. Structural priming refers to the increased likelihood of using the DOD construction following a DOD, rather than PD prime sentence.

There is extensive evidence for structural priming effects. In adults, Bock (1986) recorded 23% more PD, as opposed to DOD, targets following PD primes and 22% more DOD rather than PD targets after DOD primes. Structural priming occurs across languages, for example in German (Schenkein, 1980), Dutch (Hartsuiker and Kolk, 1998), and Japanese (Yamashita et al., 2002). Adults display abstract priming effects where primes and targets have different verbs (Bock, 1986), nouns (Cleland and Pickering, 2003), function words (Bock, 1989) or conceptual ideas (Bock and Loebell, 1990).

Structural priming is said to occur because information specified in the representation of a prime sentence is used to form the target sentence. There are however, competing models used to explain how exactly priming occurs. Proposed models include activation models (e.g., Pickering and Branigan, 1998; Kaschak and Glenberg, 2004) and priming as adaptation or implicit learning models (Chang et al., 2006; Jaeger and Snider, 2013). Taking activation models first, in their residual activation theory, Pickering and Branigan (1998) argued that hearing a DOD prime activates a lemma node representing a dative verb, a joined noun phrase, noun phrase (NP, NP) combinatorial node, and their connecting link. Structural priming occurs where speakers reuse the currently activated NP, NP node to produce another DOD construction rather than alternatively activating a joined noun phrase, prepositional phrase (NP, PP) node to produce a PD sentence. According to this account, priming can depend on abstract representations that feature information about general categories (e.g., verb) and combinatorial nodes without specifying particular words (e.g., bring). Kaschak and Glenberg (2004), on the other hand, explain structural alignment between speakers in terms of ease of memory retrieval. They argue that there is usually little additional sentence processing between the time taken for participants to comprehend primes and produce targets (i.e., participants produce targets almost immediately after hearing primes). Thus, the syntactic structure of a prime sentence is more likely to be reused in a target sentence over a suitable alternative structure because it was processed more recently and is thus easier to retrieve from memory.

Alternatively, Jaeger and Snider (2013), and Chang et al. (2006), proposed models that present priming as a process of adaptation or implicit learning. For example, in Chang et al.'s model, language users are thought to use probabilistic information based on utterances they have heard previously to make predictions about upcoming words in sentences that they read or hear. Each new input results in an updated set of associative links between representations. Error-based learning is said to occur where speakers revise a particular prediction after exposure to a sentence that fails to meet their original predictions. Chang et al. suggested that primes with less predictable properties are more salient and memorable than those with more typical features and consequently, unlike activation models, adaptation models predict increased priming from sentences that contain less frequent structures or unexpected features. There is some evidence in line with this prediction. Bock (1986) found that (infrequent) passives elicited a greater priming effect than (frequent) actives, while Peter et al. (2015) observed marginally more priming in adults (with stronger effects in children) where DOD primes contained PD-biased verbs and PD primes contained DOD-biased verbs than following primes where the prime structure matched the structural bias of the verb in question.

Another issue concerns whether structural priming effects are based purely on syntactic structure or whether lexical or semantic aspects of prime sentences such as animacy or thematic role information can also affect priming. In adults, there is evidence to suggest that sentence representations can sometimes specify lexical content. Pickering and Branigan (1998) predicted that the reactivation of any given combinatorial node with the same verb node as encountered in a prime sentence increases the likelihood of priming (i.e., when both prime and target share the same verb), termed the lexical boost (Branigan et al., 2000; Snider, 2009; Chang et al., 2012). Repetition of cognate words (Bernolet et al., 2012) and nouns (Cleland and Pickering, 2003) across primes and targets can also increase priming effects in adults. Furthermore, according to error-based learning accounts (e.g., Chang et al., 2006), learning and subsequent surprisal priming effects would be expected to occur where the sequence of words encountered in the prime is unexpected, and this might happen to a greater extent in younger children than in adults since they have had less exposure to more uncommon sentence types. Peter et al. (2015) provided supporting evidence as children showed greater increases in structural priming effects than adults for DOD prime sentences that featured PD-, as opposed to DOD-biased verbs.

However, the role of semantic information relating to thematic roles or animacy characteristics in structural priming is the subject of considerable debate. On the one hand, structural priming effects may be driven by an autonomous syntax (e.g., Bock, 1987). From this perspective, to the extent that features such as animacy might result in priming effects, these are seen as independent of any observed syntactic influence. Alternatively, priming effects may be tightly related to functionalist aspects of meaning (e.g., Bates and MacWhinney, 1982). From this perspective, semantic information is assumed to interact with syntactic information to result in increased priming effects over and above those driven purely by syntactic representations (see Pickering and Ferreira, 2008 for a review).

It is sometimes difficult to separate thematic role information from the animacy characteristics of the referents. Thus, animacy may interact with semantic role-grammatical function mappings (de Swart et al., 2008). There is clear evidence that particular syntactic structures and/or thematic roles are typically associated with particular animacy characteristics in adult speech. Experimental (Dahl and Fraurud, 1996) and corpus research (Øvrelid, 2004) demonstrates adults' preference for animate subjects and inanimate objects. Moreover, active sentences tend to contain animate agents (subjects) before inanimate patients (objects) (Bock, 1986). However, where patients are animate, adults tend to choose passives with patient subjects before agents, over actives (Gennari et al., 2012). Similarly, speakers' choices of dative structure are influenced by the animacy of the recipient/goal. Prototypical DOD sentences contain animate goals before inanimate themes and thus feature an animate-inanimate object order, whereas prototypical PD sentences have an inanimate-animate object order with inanimate themes before animate goals (Bresnan et al., 2007). Bresnan et al. (2007) and Bresnan and Hay (2008) modeled English adult corpus data and found that PD sentences are preferred where recipients are inanimate, whereas DOD sentences are preferred where recipients are animate.

Thus, speakers' representations of particular sentence types appear closely related to the thematic roles and the noun animacy characteristics that typically appear in them. These properties are often highly correlated in prototypical sentences. For example, the prime PD sentence: *the girl brought the ball* [inanimate theme] *to the monkey* [animate goal] maps onto the prototypical inanimate-animate noun ordering with an inanimate theme and animate goal associated with the PD structure. This means that priming effects, when describing a similar scene, could be based on abstract syntactic representations, but could also rely on thematic roles (e.g., theme before goal) or noun animacy (e.g., inanimate before animate). It is therefore important to examine priming effects for both prototypical and non-prototypical primes and targets to ascertain the extent to which syntactic effects are independent of semantics. For example, prototypical noun animacy and thematic roles do not always co-occur, as shown by the non-prototypical target PD sentence: *the boy brought the tiger* [animate theme] *to the zoo* [inanimate goal]. Structural priming, in this instance, cannot depend on the repetition of animacy noun orders because the animate-inanimate order in the target is typically associated with DOD sentences (e.g., *the boy brought the* *monkey* *a* *ball*) rather than that which occurs in the prototypical PD prime. Structural priming must rather rely on the repetition of the abstract syntactic frame.

There is evidence to suggest that thematic roles do not tend to interact with syntax to influence structural priming in adults. Bock and Loebell (1990) for instance, showed that participants produced more passive targets to describe transitive scenes following both passive and locative primes as compared to active primes. Both their passive (e.g., *the construction worker was hit by the bulldozer*) and locative primes (e.g., *the construction worker was digging by the bulldozer*) included a by-phrase, but they differed in the thematic role assigned to the first noun (patient vs. agent). Interestingly, the magnitude of priming did not differ between the two prime types, demonstrating that participants were not primed to map agents to the first noun to produce active targets but rather repeated the structural by-phrase to produce *patient-agent* passives.

However, there is the suggestion that at least under some circumstances, thematic role assignment may play a direct role in priming independently of syntax. Chang et al. (2003) argued that thematic role information may be specified in representations used to facilitate priming where thematic role distinctions are necessary to distinguish between two kinds of structures, for example where primes contain the same syntactic structure (e.g., a noun phrase—verb phrase—prepositional phrase locative construction) but different thematic role orders (e.g., theme-location [*the man sprayed water on the wall*]/location theme [*the man sprayed the wall with water*]). They found that speakers were primed to reuse either theme-location or location-theme role orders, independent of the syntactic structure of the prime, arguing that because the influence of thematic roles may be weaker than the effects of syntactic structure, it can only be observed in adult speakers in instances where syntactic structure decisions are neutralized (see also Hare and Goldberg, 1999; Chang et al., 2006).

Turning to animacy effects on structural priming, studies in which researchers have manipulated animacy cues suggest that animacy does not interact with syntax to impact structural priming in adults, although animacy can have an independent influence. Bock (1986) found that the magnitude of priming of passives was not dependent on whether the prime contained a human or non-human agent or patient. Likewise, Bock et al. (1992) observed no interaction between animacy and syntax in the structural priming of actives and passives, irrespective of whether primes contained animate subjects and inanimate objects or inanimate subjects and animate objects. Similarly, Huang et al. (2016) found no difference in the magnitude of structural priming of dative sentences in Mandarin as a function of whether the recipient was animate or inanimate.

However, additional research is required before we can confidently conclude that animacy does not interact with syntax to result in priming. For example, Huang et al.'s (2016) experiment using datives manipulated the animacy of the recipient but kept all themes inanimate. This means that non-prototypical primes had neither animacy contrasts between themes and recipients nor a noun animacy order cue corresponding to either prototypical DOD or PD sentences. Thus, although it appears that the animacy of the recipient alone cannot influence priming, this leaves open the possibility that a combination of semantic cues might have an effect. Thus, the first goal of the present study was to ascertain whether adults might show differential priming effects with dative constructions as a function of whether the animacy-semantic role cues in the prime sentence and target scene are prototypical or non-prototypical.

Although the current evidence suggests that thematic role and animacy information has relatively little impact on priming in adults (pending confirmatory evidence from tightly controlled studies), there is reason to believe that the situation may be different in children. Usage-based approaches to the development of linguistic representations during first language acquisition argue for the gradual development of abstract grammatical structures which emerge from specific exemplars that children have experienced. This means that initial representations might be tied to the specific sentence-level properties of high frequency events, including information about their prototypical semantic roles and animacy characteristics (e.g., Ambridge et al., 2015). There is considerable evidence to support this claim. Children perform better in sentence comprehension and production with sentences with prototypical animacy mappings. For instance, 2 year olds interpret active sentences more accurately when they contain animate agents and inanimate patients as opposed to inanimate agents and animate patients (Chan et al., 2009, see also Corrigan, 1988), 3 and 4-year-olds are better able to produce and comprehend object relative clauses with inanimate rather than animate head nouns (Kidd et al., 2007; Brandt et al., 2009), and from around 3 years, children are better able to produce passives with animate rather than inanimate patients (Lempert, 1989, and in priming in 5 and 6 year olds, Vasilyeva and Waterfall, 2012, see also Cook, 1976 for animacy effects on comprehension of datives). Thus, one intriguing possibility is that children's sentence representations may be more closely tied to semantics than those of adults, and therefore children may show (greater) semantic effects in structural priming that would provide information about the nature of their underlying representations.

To date, evidence on the nature of priming in children is mixed. Structural priming has been found to occur in children (e.g., Huttenlocher et al., 2004) in the absence of lexical overlap, with robust evidence for abstract priming across verbs. Where primes and targets featured different verbs, 3 and 4 year old children have been shown to demonstrate abstract structural priming in both English sentence production (Rowland et al., 2012; Peter et al., 2015) and comprehension (Thothathiri and Snedeker, 2008). There is, however, conflicting evidence regarding whether or not children show abstract priming across nouns. In Peter et al.'s (2015) study, children showed dative priming where primes and targets contained different nouns, whereas in contrast, Savage et al. (2003), found no priming of active and passive sentences in 3 and 4 year olds where primes and targets had different subject and object nouns. The discrepancy in results may be due to methodological differences between the two studies. In Savage et al.'s (2003) study, children had to repeat the entire prime sentence and produce full target sentences for responses to be included whereas Peter et al. (2015) did not require participants to repeat primes and they only produced the post-verbal part of the sentence. Children tested by Savage et al. (2003) may have been less likely to show evidence of structural priming effects in the absence of noun overlap due to the high cognitive demands and strict coding scheme.

Typically, studies testing whether children demonstrate structural priming, and those manipulating lexical overlap between primes and targets, have ignored the potential role of semantic overlap between primes and targets in terms of thematic roles or the animacy of the nouns. As a result, it is currently unclear whether structural priming in children is always driven by syntax alone, or if there are interactions between semantic and syntactic representations which can influence the magnitude of the priming effect. Studies manipulating prime and target animacy cues in relation to specific thematic roles are consequently needed to identify whether semantic processes facilitate structural priming.

However, there is some limited evidence to suggest that semantic factors may result in stronger priming effects in children than those observed in the absence of semantic overlap, although when semantic overlap influences the magnitude of priming depends on the semantic factor studied. Goldwater et al. (2011) found that the magnitude of dative priming in 4 year olds was significantly increased where primes conveyed conceptually similar, as opposed to dissimilar messages to that of the target scene: the DOD prime sentence *the girl is telling her classmates a story*, (semantically similar) was more likely to yield a DOD target than *the boy is throwing the catcher a baseball* (semantically dissimilar) where the target scene depicted a teacher showing a book to her students. On the other hand, there was no effect of conceptual similarity on priming in 5 year olds. In contrast, Vasilyeva and Gámez (2015) found that the magnitude of priming of passives in 5 year olds was increased where primes and targets both contained animate patients/inanimate agents as opposed to instances where targets contained animate patients/inanimate agents while primes contained inanimate patients/animate agents. However, it is unclear whether these effects are robust due to the small number of participants tested to assess the extent to which the observed noun animacy effects reflected the properties of the prime independent of the properties of the target scene.

To conclude, to date, there is somewhat mixed evidence on the possible role of semantic information in structural priming across development due to a combination of a lack of tightly controlled studies, relatively small sample sizes, or a lack of developmental comparisons. However, one possibility is that children's early linguistic representations might be more tightly linked to the prototypical instantiations of the structures they have experienced than those of older children and adults. As a result, further research is necessary to decide if and when semantic role and animacy effects can be observed and how any observed effects change developmentally. Children typically perform better in comprehension and production with prototypical exemplars of specific sentence types, which might lead us to predict that they will show greater priming effects for sentences containing prototypical animacy-semantic role mappings for the syntactic structure concerned. Nevertheless, usage-based approaches to language acquisition would predict semantic effects to decrease over development due to increasing abstraction and connectivity across children's linguistic systems, as suggested, for example, by older children's reduced reliance on animacy to interpret a range of sentence structures (e.g., Corrigan, 1988; Brandt et al., 2009; Chan et al., 2009). Thus, a second goal of the present study was to determine whether animacy-semantic role mappings interact with syntax to influence structural priming of datives in children, and whether this changes developmentally.

One final question we explore in this paper is whether animacy cues can exert an influence on target word orders independently of (PD/DOD) syntactic structure. There is some evidence that speakers tend to produce sentences with animate nouns before inanimate nouns, regardless of the particular syntactic structures or thematic roles involved. For instance, adults (Bock, 1986; Gennari et al., 2012) and children (Bloom, 1970; Dewart, 1979; Lempert, 1989) have been shown to alternate between active and passive constructions in such a way as to maintain an animate-inanimate noun order. Moreover, children around 2 years of age have been found to demonstrate a “first-noun-as-agent” interpretation of unfamiliar syntactic structures including the conjoined agent intransitive (e.g. children sometimes interpret the sentence “*Bunny and Duck are meeking*”—a nonce verb—to mean that Bunny is carrying out a causal action on Duck), and their ability to interpret conjoined agent intransitive structures is partly determined by the animacy characteristics of the two pre-verbal nouns (Noble et al., 2016). This has been explained in terms of children extracting sentence-general information about the nature of particular arguments (e.g., pre-verbal nouns are often animate and agents) and applying this information when processing unfamiliar sentence types (Chang et al., 2006).

There is some evidence from adult studies that priming can occur at the level of noun animacy, independently of syntactic structure. Bock et al. (1992) found that following active and passive primes containing inanimate pre-verbal subjects before animate post-verbal objects, participants were more likely to describe target scenes with an inanimate agent and animate patient using active sentences, mirroring the *inanimate-animate* ordering of the primes, irrespective of their syntactic structure (see also Kempen and Harbusch, 2004). For children, however, empirical evidence is lacking, and for both groups it is unclear if effects of noun animacy will be attested for less frequently encountered post-verbal contrasts. It is possible that children and adults may be sensitive to the animacy characteristics of post-verbal nouns in datives, independently of the syntactic structure (PD or DOD) in which they appear. We therefore ask whether the ordering of two post-verbal nouns in datives and their associated animacy characteristics can influence subsequent sentence production independently of the semantic role instantiated in the prime.

We carried out a priming study using PD and DOD primes and target scenes with 3 year olds, 5 year olds and adults, and conducted two sets of analyses to investigate whether animacy cues can affect sentence processing both through interactions with syntax and independently of it. In Analysis one, we investigated whether: (i) structural priming of datives fundamentally relies on the repetition of abstract syntactic representations (ii) animacy influences the magnitude of structural priming in children and adults where the animacy of both themes and goals is systematically manipulated, and (iii) any observed animacy effects on priming change over the course of development. Since prior research seems to imply relatively strong interactions between animacy and syntax and that these play a greater role in children's sentence processing, we tested the following hypotheses: (i) structural priming effects will be greater where primes have prototypical animacy cues, (ii) priming will be greater where primes and targets have matching animacy-semantic role mappings—consistent with Vasilyeva and Gámez (2015), (iii) the relative increase in priming effects where animacy-semantic role mappings are prototypical and matching across primes and target pairs will decrease with age.

In Analysis two we sought to obtain insight into how speakers might construct sentences based on learned ordering of animate and inanimate nouns in sentences by assessing whether animacy priming effects occur independently of structural priming and semantic role assignment. Since previous research indicates that children appear to be sensitive to animacy-semantic role mappings (e.g., Gertner and Fisher, 2012; Vasilyeva and Gámez, 2015) and that reliance on semantic content decreases with age (Corrigan, 1988) we tested the following hypotheses: (i) noun animacy priming effects will be stronger where primes and targets have prototypical semantic-role mappings, (ii) noun animacy priming effects will be greater where primes and targets have matched as opposed to mismatched animacy-semantic role mappings, and (iii) the relative increase in priming effects due to prototypicality and prime-target match will decrease with age.

## Materials and methods

### Participants

We tested 143 participants; 47 three year olds (24 females), 48 five year olds (25 females), and 48 adults (35 females). One 3 year old was excluded for their failure to produce any dative sentences. Participants were monolingual British English speakers without any reported language or developmental problems. Children were recruited from, and tested in their schools and nurseries in Manchester while adult participants were students and staff recruited from the University of Manchester and were tested in a laboratory on campus.

### Ethical guidelines

This study was conducted with ethical approval from the University of Manchester's Research Ethics Committee and the researcher had Disclosure and Barring Service (DBS) clearance. All adult participants and caregivers acting as proxy consenters for children gave written consent while all child participants gave verbal assent.

### Visual stimuli

**Figure 1:**
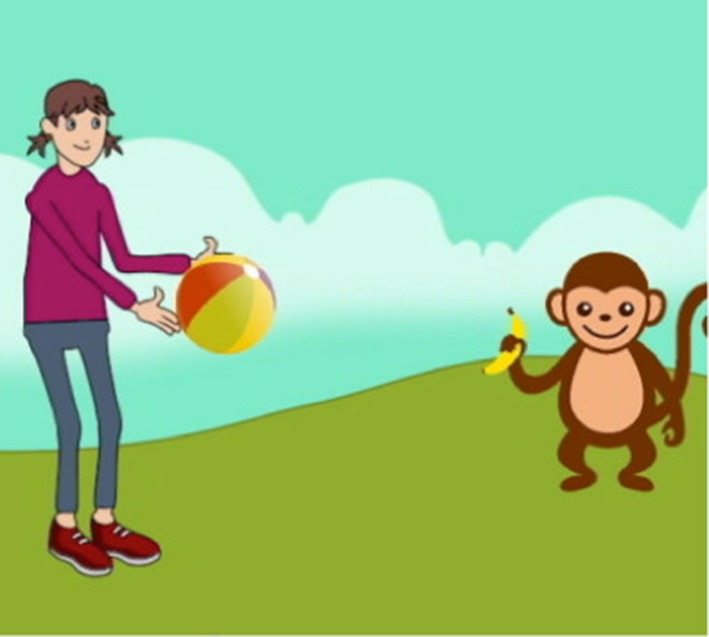
Prime animation: The girl brought the monkey a ball (DOD)/The girl brought a ball to the monkey (PD).

Sixty-eight, 10-s animations were created in *Anime Studio Pro 10* and presented on a laptop using Microsoft PowerPoint. Forty-eight animations (24 for primes and 24 for targets) portrayed ditransitive events (e.g., a girl bringing a monkey a ball). In each animation, the full transfer action was shown in the first 5 s. The remaining 5 s showed the subject holding the theme and moving toward the goal once more without showing the transfer action. For example, the animation for *the girl brought the monkey a ball* depicted a girl bringing a ball to a monkey and leaving it there. The girl then returned with the ball to her starting position and began moving toward the monkey again without actually reaching the monkey or transferring the ball this second time. The direction of transfer was left to right. Events involved agents either; throwing, bringing, sending, giving, handing or showing themes to goals. See Figure 1 for an example animation.

Twenty animations depicted intransitive events featuring two characters simultaneously acting in the center of the screen (e.g., a boy and girl jumping). Eight of these were used as practice scenes (four each for the experimenter and participant) and 12 were used as fillers (six each). As the experiment involved a bingo game, 48 bingo cards were created to correspond with 23 prime and 24 target scenes and 1 filler animation. Each bingo card featured a screen shot image of an animation. Two 2 × 3 rectangular bingo grid cards were used to record matches.

### Sentence stimuli

Eighty-two sentences were created as descriptions for the 68 animations. These included:

- Practice Items (4): present-tense intransitive sentences for the experimenter's turn in practice trials to introduce participants to the task.
- Fillers (6): present-tense intransitive sentences for the experimenter's turn in filler trials to limit priming effects across prime-target pairs.
- Primes (48): past tense dative sentences which included 24 DOD and 24 PD counterparts corresponding to the 24 prime scenes. Six different prime sentences were assigned to each of the four experimental conditions.
- Targets (24): Six different verbs were included in sentence initiations for target sentences (e.g. *the boy brought*). Primes and targets always contained the same verb and participants completed these sentence initiations to produce the full target sentence. See Table 1 for example prime sentences and target elicitation scenes.

**Table 1:** Example prime sentences and target elicitation scenes for each condition.

**Condition**	**DOD Prime**	**PD Prime**	**Target Elicitation Scene**
Prototypical Prime (AN goal & IN theme) / Matched Target	*The girl brought the monkey a ball*	*The girl brought a ball to the monkey*	Transfer of a flower from boy to a snail
Prototypical Prime (AN goal & IN theme) / Mismatched Target	*The girl brought the bee a flower*	*The girl brought a flower to the bee*	Transfer of a monkey from a boy to a zoo
Non-prototypical Prime (AN theme & IN goal)/ Matched Target	*The girl brought the zoo a tiger*	*The girl brought a tiger to the zoo*	Transfer of a bee from a boy to a zoo
Non-prototypical Prime (AN theme -& IN goal) / Mismatched Target	*The girl brought the garden a snail*	*The girl brought a snail to the garden*	Transfer of a ball from a boy to a tiger

The subject/agent was always a girl in primes and a boy in target sentences while animate themes and goals were non-human animals (e.g., lion, frog, and cow). Themes and goals for primes and targets were chosen from one list of 24 non-human animate objects, and another list of 24 inanimate objects (e.g., ball, rope, farm, and zoo), each divided into four blocks. All nouns were selected to be familiar to young children. One animate and inanimate object was assigned to each sentence. Where animacy-semantic role mappings needed to be prototypical, animate objects were assigned to goals and inanimates to themes. The reverse was done for non-prototypical mappings. Primes and targets within the same pair always contained different themes and goals. See Table 2 for clarification.

**Table 2:** Order of animate (AN) and inanimate (IN) nouns in prime and target sentences.

**Condition**	**Prime**	**Target**
Prototypical Prime (AN goal & IN theme)/Matched Target	AN block 1, IN block 1	AN block 2, IN block 4
Prototypical Prime (AN goal & IN theme)/Mismatched Target	AN block 2, IN block 2	AN block 3, IN block 1
Non-prototypical Prime (AN theme & IN goal)/Matched Target	AN block 3, IN block 3	AN block 4, IN block 2
Non-prototypical Prime (AN theme & IN goal)/Mismatched Target	AN block 4, IN block 4	AN block 1, IN block 3

*Threw, brought, gave, handed, showed*, and *sent* were the six alternating dative verbs used. Each verb occurred once within each of the four conditions. To make the experiment easier for children, each noun was only ever associated with one verb (e.g., *ball* only occurred with *brought*). While the blocks within the object lists were rearranged, the order of objects within each block was not. Primes and targets within the same pair contained the same verb to control for the possibility that structural priming is facilitated by lexical overlap (Savage et al., 2003; Chang et al., 2012). We sought to increase the likelihood of structural priming effects occurring since a basic priming effect is required in order to identify whether priming is driven by abstract representations or representations specifying animacy-semantic role mappings. To limit confounding effects of sentence length on priming effects, the mean number of syllables in each sentence and across conditions was controlled as much as possible.

### Procedure

The 20 min experiment used Rowland et al.'s (2012) bingo game paradigm. The experimenter and participant sat side-by-side, facing a laptop and each held a bingo grid card. Participants were given six Bingo cards, each corresponding to a target scene, while the experimenter had five cards portraying different prime scenes and one depicting the final filler scene. The experimenter played the animations, beginning with four *practice-practice* trials, followed by alternating *prime-target* and *filler-filler* trials.

**Figure 2:**
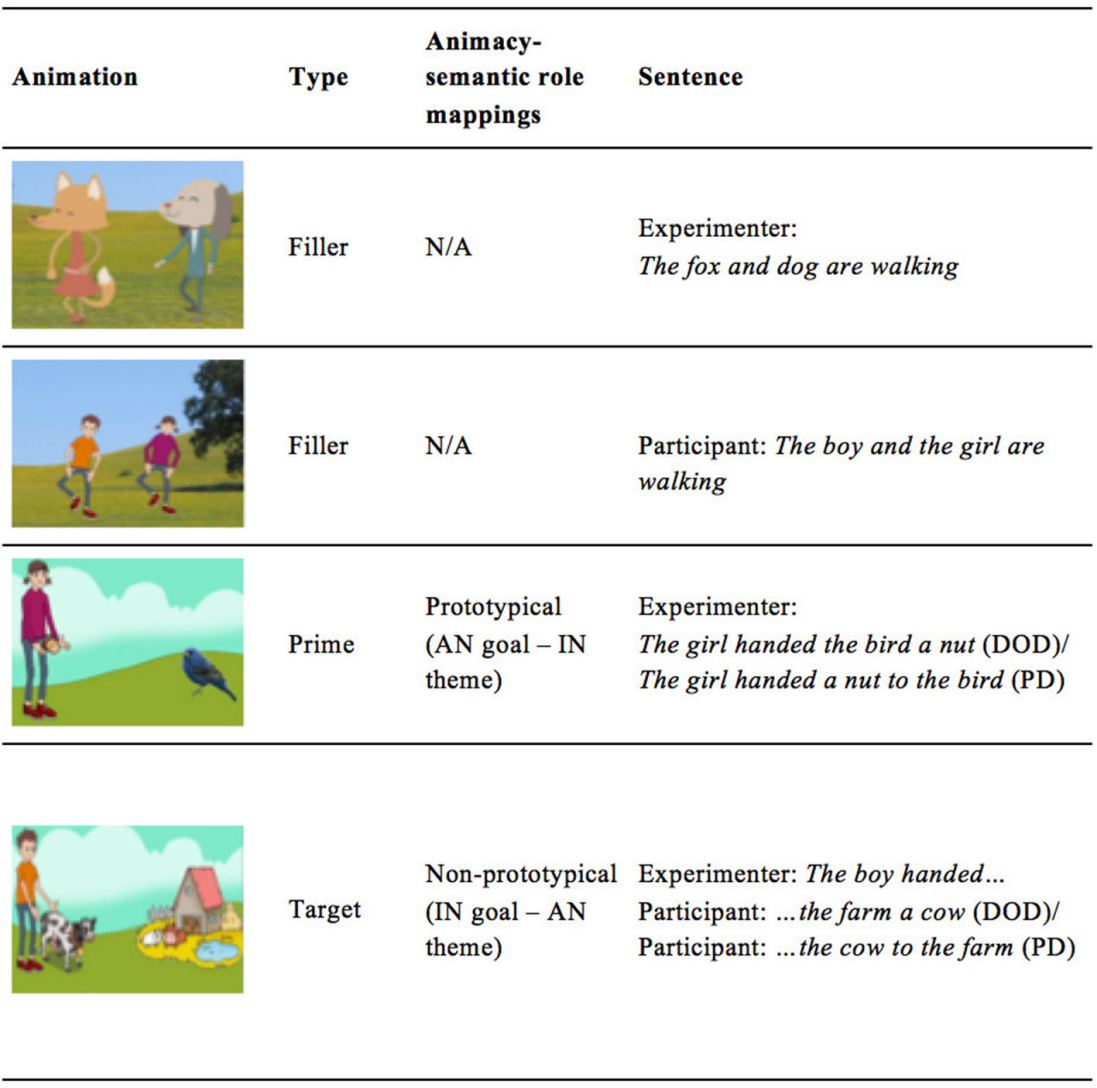
Protocol for filler and priming trials.

The experimenter described the first scene and produced the first sentence in each pair, producing all primes while participants described the second scene in each pair, including targets. On target trials, the experimenter produced sentence initiations (e.g., *the girl brought*) to encourage participants' use of datives. Participants formed their own target structures as they finished the sentence (e.g., *the monkey a ball* or *the ball to the monkey*). See Figure 2 for the experimental protocol.

When the experimenter and participant encountered a scene that corresponded with one of their bingo cards, they placed this card on one of their Bingo grid boxes. The winner was the first to fill all six boxes on their grid; this was always the participant. Adults were tested in the same way as children although they did not participate in a bingo game. While the experimenter always produced the subject and verb for children's target sentences, adults often produced entire target sentences. This occurred wherever adults did not wait for the experimenter to produce the initial part of target sentences but were rather quick to produce the full target sentence (including the subject and verb) without prompting.

## Analysis 1: structural priming

### Design

We used a 3 × 2 × 2 × 2 mixed design. Age (3 years/5 years/adults) and prime structure (double-object dative [DOD]/prepositional dative [PD]) were between-subject independent variables. Prime animacy-semantic role mappings (prototypical [AN goal & IN theme]/ non-prototypical [AN theme & IN goal] and prime-target match in animacy-semantic role mappings (match/mismatch) were within-subjects independent variables. The production of DOD target responses was our dependent variable.

### Coding

Target responses were coded for syntactic structure (double-object dative [DOD], prepositional dative [PD], and OTHER). Only DOD and PD target sentences were included in the analyses. Participants had to produce each target response in one turn, regardless of whether the experimenter had to prompt them to speak or define an object when asked. Target responses that did not contain the initial verb were included (e.g., *the ball to the monkey*). Objects seen in target scenes had to be accurately labeled and assigned to semantic roles. Targets with incorrectly named nouns were acceptable where this was a result of a vocabulary error and not a misunderstanding of the event shown in the target scene. For example, where the target scene showed the transfer of a ball between a boy and tiger, the response *the boy brought the lion/cat a ball* was acceptable but not *the boy brought the mouse a ball*. Participant responses were coded as follows:

1. DOD: sentences with a *goal—theme* structure (e.g., *the boy brought the tiger a ball*).
2. PD: sentences with a *theme – preposition - goal* structure (e.g. *the boy brought a tiger for the monkey*). Both *to* and *for* were suitable prepositions.
3. OTHER: Such responses were excluded from the analyses and included:
  - Sentences without a DOD or PD structure (e.g., intransitive and/or incomplete sentences with only one noun such as *the boy threw the whale*, or locatives such as *the boy threw the whale into the sea*).
  - Incomplete sentences with one object and a preposition but no second object (e.g., *the boy threw the food to*).
  - Sentences where nouns were assigned to the wrong semantic role (e.g., *the boy brought the ball* [goal] *a tiger* [theme], where the target scene actually showed the transfer of a ball [theme] between a boy and tiger [goal]. A misunderstanding of the target scene may influence target structures where animacy cues might interact with syntactic structures.
  - Sentences with incorrectly named nouns, indicating the participant's misunderstanding of the event shown in the target scene (e.g., *the boy brought the zoo/mouse a ball* instead of *the boy brought the tiger a ball*).

The percentage of OTHER target responses was 38% in the 3 year old group, 28% in 5 year olds and 27% in the adults. This is to be expected because although our events involved three participants, it is perfectly acceptable to focus on only a subset of these in a linguistic description of the scenes.

### Results

We examined whether structural priming is facilitated by interactions between animacy cues and syntactic structures. We assessed the extent to which the magnitude of this priming effect was dependent on whether (i) animacy-semantic role mappings are prototypical or non-prototypical and (ii) there is a match or mismatch in animacy-semantic role mappings across prime sentences and target scenes. We also examined whether there were any developmental changes in priming effects.

**Figure 3:**
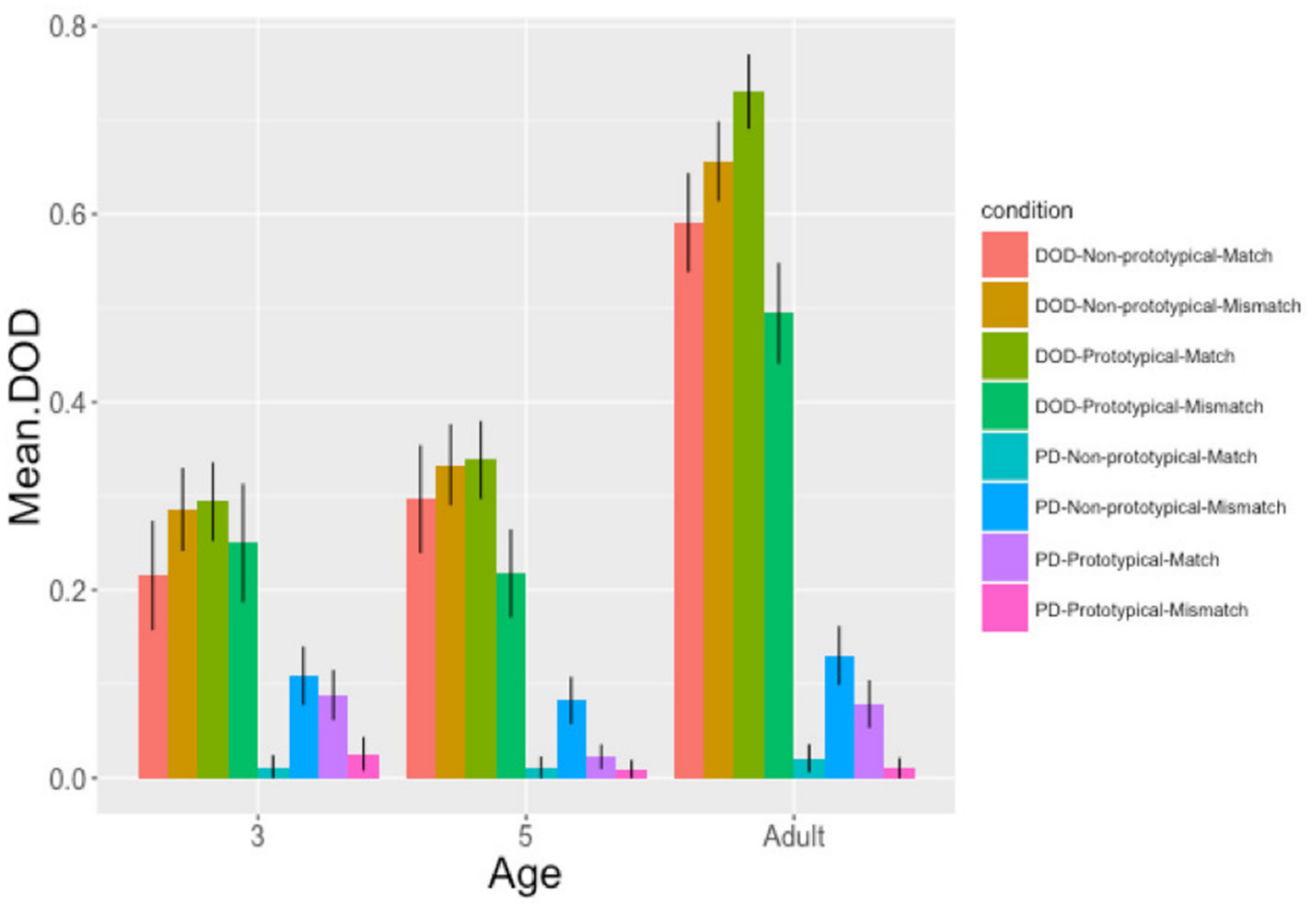
The mean proportion of DOD responses following DOD and PD primes where primes contained either prototypical or non-prototypical animacy-semantic role mappings and these mappings were either matched or mismatched across primes and targets (SE in error bars).

The data were analyzed using logistic mixed effects models in R, using the glmer function of the lme4 package (lme4 version 1.1-11: R Core Team, 2012). Fixed effects included: age (3 years = −1; 5 years = 0; adult = 1), prime animacy-semantic role mappings (prototypical [AN theme − IN goal] = 1; non-prototypical [IN theme − AN goal] = 0) and prime-target match in animacy-semantic role mappings (match = 1; mismatch = 0). All variables were centered to reduce multicollinearity (Neter et al., 1985). Random effects entered into the models were participant and item, although final models included only participant as models with item as a random effect failed to converge. Interaction terms were added to the models and retained if they improved model fit, as determined by ANOVA comparisons. The analyses were separated by age since models fitted to the full data set which included interactions with age did not converge. Within each age group, further models were run to explore each significant interaction effect using the Bonferroni correction and all *p*-values for individual predictors were obtained from the model summary output. Finally, where the results indicated a difference between children and adults, models comparing the 3 year olds and adults were run to confirm the presence of interactions between the manipulated sentence-level variables and age. The mean proportion of DOD target responses produced in each experimental condition is shown in Figure 3.

#### Age three

The model initially contained only main effects of prime structure, prime animacy-semantic role mappings and prime-target match, but was significantly improved by adding a three-way interaction term (*p* = 0.03). We found a significant main effect of prime structure β = 2.46 (*SE* = 0.54), *z* = 4.55, *p* < 0.001 whereby more DOD targets were produced following DOD (*M* = 0.27, *SE* = 0.02) as opposed to PD primes (*M* = 0.06, *SE* = 0.01) and a significant three-way interaction between prime structure, prime animacy-semantic role mappings and prime-target match β = 2.21 (*SE* = 0.75), *z* = 2.95, *p* = 0.003. See Table 3 for these model results.

**Table 3:** Mixed effects logistic model results for children aged 3.

**Fixed Effects**	**Estimate**	**Standard Error**	***z*-value**	***p*-value**
Intercept	−2.45	0.28	−8.65	0.001^**^
Prime Structure	2.46	0.54	4.55	0.001^**^
Prime Animacy-Semantic Role Mapping	0.17	0.37	0.47	0.641
Prime-Target Match	−0.31	0.37	−0.84	0.402
Prime Structure ^*^ Prime Animacy-Semantic Role Mapping	0.07	0.75	0.09	0.925
Prime Structure ^*^ Prime-Target Match	0.36	0.75	0.47	0.627
Prime Animacy-Semantic Role Mapping ^*^ Prime Target Match	2.21	0.75	2.95	0.003^**^
Prime Structure ^*^ Prime Animacy-Semantic Role Mapping ^*^ Prime Target Match	−3.17	1.50	−2.12	0.03^*^

* *p < 0.05*.

** *p < 0.001*.

In order to interpret this three-way interaction, another model was fitted for each level of prime structure (DOD and PD). The Bonferroni method was used with a corrected alpha level of 0.025 for all *post hoc* analyses. Analysis of DOD primes failed to reveal any significant effect for prime animacy-semantic role mappings, β = 0.20 (*SE* = 0.31), *z* = 0.65, *p* = 0.518, prime-target match, β = −0.12 (*SE* = 0.31), *z* = −0.40, *p* = 0.688, or the interaction between the variables, β = 0.61 (*SE* = 0.61), *z* = 1.02, *p* = 0.31. Analysis of PD primes, however, revealed a significant two-way interaction between prime animacy-semantic role mappings and prime-target match, β = 3.89 (*SE* = 1.39), *z* = 2.81, *p* = 0.005.

Two further models were run for PD primes, one for each level of animacy-semantic role mapping (prototypical [AN goal & IN theme]/non-prototypical [AN theme & IN goal]). Where PD primes featured non-prototypical animacy-semantic role mappings, there was a marginally significant effect of prime-target match, β = −2.33 (*SE* = 1.07), *z* = −2.19, *p* = 0.029. Fewer DOD responses were produced where targets contained matched (non-prototypical) animacy-semantic role mappings (*M* = 0.01, *SE* = 0.03) as opposed to mismatched (prototypical) animacy-semantic role mappings (*M* = 0.11, *SE* = 0.03). However, where PD primes contained prototypical animacy-semantic role mappings there was no significant effect of prime-target match, β = 1.47 (*SE* = 0.85), *z* = 1.73, *p* = 0.08.

To summarize, 3 year olds showed a significant structural priming effect, producing more DOD targets, following DOD primes as compared to PD primes. While animacy-semantic role mappings had no effect on DOD sentence priming, they had a marginal influence on the magnitude of PD sentence priming. Three year olds produced slightly more PD targets following PD primes where prime sentences contained non-prototypical animacy-semantic role mappings (AN theme & IN goal) and target scenes contained matching (non-prototypical [AN theme & IN goal]), as opposed to mismatching (prototypical [AN goal − IN theme]) animacy-semantic role mappings.

#### Age five

The model originally featured only main effects but was significantly improved by adding two-way interaction terms between the variables (*p* = 0.007). There was a significant main effect of prime structure β = 2.99 (*SE* = 0.50), *z* = 6.00, *p* < 0.001 whereby more DOD targets were produced following DOD (*M* = 0.30, *SE* = 0.02) as opposed to PD primes (*M* = 0.02, *SE* = 0.01) and a significant two-way interaction between prime animacy-semantic role mapping and prime-target match β = 3.19 (*SE* = 0.60), *z* = 5.29, *p* = <0.001. See Table 4 for these model results.

**Table 4:** Mixed effects logistic model results for children aged 5.

**Fixed Effects**	**Estimate**	**Standard Error**	***z*-value**	***p*-value**
Intercept	−2.60	0.27	−9.78	001^**^
Prime Structure	2.99	0.50	6.00	001^**^
Prime Animacy-Semantic Role Mapping	−0.57	0.35	−1.64	0.100
Prime-Target Match	−0.22	0.35	−0.63	0.526
Prime Structure ^*^ Prime Animacy-Semantic Role Mapping	0.67	0.70	0.96	0.338
Prime Structure ^*^ Prime-Target Match	0.95	0.71	1.35	0.176
Prime Animacy-Semantic Role Mapping ^*^ Prime Target Match	1.24	0.51	2.43	0.015^*^

* *p < 0.05*.

** *p < 0.001*.

To interpret the two-way interaction a model was fitted for each level of prime animacy-semantic role mapping (prototypical [AN goal & IN theme]/non-prototypical [AN theme & IN goal]). The Bonferroni method was used with a corrected alpha level of 0.025 for all post hoc analyses. For prototypical prime animacy-sematic role mappings there was a significant effect of prime-target match, β = 0.83 (*SE* = 0.35), *z* = 2.35, *p* = 0.018. DOD production was higher where targets featured matched (prototypical; *M* = 0.18, *SE* = 0.02) as opposed to mismatched (non-prototypical; *M* = 0.09, *SE* = 0.02) animacy-semantic role mappings. However, where primes contained non-prototypical animacy-semantic role mappings there was no effect of prime-target match, β = −0.48 (*SE* = 0.36), *z* = −1.35, *p* = 0.177. There was no difference in the production of DOD targets where targets featured matched (non-prototypical; *M* = 0.14, *SE* = 0.03) as compared with mismatched (prototypical; (*M* = 0.09, *SE* = 0.03) animacy-semantic role mappings.

To summarize, there was a significant structural priming effect in 5 year olds, as they produced more DOD targets, following DOD primes as compared to PD primes. The magnitude of structural priming effects was not influenced by whether animacy-semantic role mappings were prototypical (AN goal & IN theme) or non-prototypical (AN theme & IN goal) and/or matched or mismatched across primes and targets. However, animacy-semantic role mappings did to some extent, influence 5 year olds' DOD target production, regardless of prime structure. Where prime animacy-semantic role mappings were prototypical (AN goal & IN theme) they produced more DOD responses where targets contained matched prototypical, as opposed to mismatched non-prototypical mappings.

#### Adults

The model originally featured only main effects but was significantly improved by adding two-way interaction terms between the variables (*p* < 0.001). We found a significant effect of prime structure β = 6.53 (*SE* = 1.28), *z* = 5.13, *p* < 0.001 with more DOD targets produced following DOD (*M* = 0.64, *SE* = 0.02) as opposed to PD primes (*M* = 0.07, *SE* = 0.12) and a significant two-way interaction between prime animacy-semantic role mapping and prime-target match β = 3.19 (*SE* = 0.60), *z* = 5.29, *p* < 0.001. The Bonferroni method was used with a corrected alpha level of 0.025 for all post hoc analyses. See Table 5 for these model results.

**Table 5:** Mixed effects logistic model results for adults.

**Fixed Effects**	**Estimate**	**Standard Error**	***z*-value**	***p*-value**
Intercept	−2.32	0.60	−3.85	0.001^**^
Prime Structure	6.53	1.28	5.13	0.001^**^
Prime Animacy-Semantic Role Mapping	−0.21	0.34	−0.599	0.549
Prime-Target Match	0.30	0.35	0.874	0.382
Prime Structure ^*^ Prime Animacy-Semantic Role Mapping	0.21	0.69	0.30	0.763
Prime Structure ^*^ Prime Target Match	1.14	0.69	1.64	0.100
Prime Animacy-Semantic Role Mapping ^*^ Prime Target Match	3.19	0.60	5.29	0.001^**^

** *p < 0.001*.

To interpret the two-way interaction, another model was fitted for each level of prime animacy-semantic role mapping (prototypical [AN goal & IN theme]/non-prototypical [AN theme & IN goal]). For primes with prototypical animacy-sematic role mappings we found a significant effect of prime-target match, β = 2.608 (*SE* = 0.51), *z* = 5.09, *p* < 0.001. DOD production was higher where targets featured matched (prototypical; *M* = 0.43, *SE* = 0.03) as opposed to mismatched (non-prototypical; (*M* = 0.25, *SE* = 0.03) animacy-semantic role mappings. Where primes contained non-prototypical animacy-semantic role mappings, there was also a significant effect of prime-target match β = −1.33 (*SE* = 0.43), *z* = −3.12, *p* < 0.001. Fewer DOD responses were produced where targets contained matched (non-prototypical; *M* = 0.30, *SE* = 0.03) as opposed to mismatched (prototypical; (*M* = 0.40, *SE* = 0.03) animacy-semantic role mappings.

To summarize, adults showed a significant structural priming effect, producing more DOD targets following DOD primes as compared to PD primes. The magnitude of structural priming effects was not at all influenced by whether animacy-semantic role mappings were prototypical (AN goal & IN theme) or non-prototypical (AN theme & IN goal) and/or matched or mismatched across primes and targets. However, animacy-semantic role mappings did influence adults' DOD sentence production, independently of prime structure. They produced more DOD responses where target scenes contained prototypical animacy-semantic role mappings regardless of whether these mappings matched with those in prototypical (AN goal & IN theme) primes or mismatched with non-prototypical (AN theme & IN goal) primes.

### Overall summary

All age groups showed an effect of structural priming, producing more DOD responses following DOD primes, as compared to PD primes, although the size of the priming effect was larger in the adults (57%) than in the children (21 and 28% in the 3 and 5 year olds respectively), in line with the enhanced priming driven by verb overlap between prime and target seen in adults in previous studies (Rowland et al., 2012). Three year olds also exhibited effects of animacy-semantic role mappings on the magnitude of structural priming, showing a marginal increase in PD sentence priming effects where primes and targets contained matching non-prototypical (AN theme & IN goal) (although no effects were observed for DOD primes). However, there were no animacy effects on the magnitude of structural priming in 5 year olds or adults. To confirm these apparent developmental differences between 3 year olds and adults, two models were run to test for an interaction between age, prime animacy-semantic role mapping and prime-target match for each level of prime structure (DOD/PD). This revealed a significant interaction for DOD but not PD primes, indicating that 3 year olds, but not adults, were influenced by animacy information in relation to specific prime structures. On the other hand, animacy-semantic role mappings influenced DOD target production in 5 year olds and adults, independently of prime structure. Both age groups exhibited an increased production of DODs for targets with prototypical animacy-semantic role mappings (AN goal & IN theme) when the target mappings matched with prototypical (AN goal & IN theme) primes. Only adults showed this increase in DOD production when targets with prototypical mappings (AN goal & IN theme) followed mismatching non-prototypical (AN theme & IN goal) primes.

### Interim discussion

Our results show a main structural priming effect in all age groups, consistent with findings from previous studies on 3 and 5 year olds (Rowland et al., 2012; Peter et al., 2015) and adults (Bock, 1986) while also providing further insight into how priming works. We found that structural priming relies, first and foremost, on the repetition of syntactic structures and not animacy-semantic role ordering, at least in the case of PD and DOD structures. This was initially unclear due to methodological issues with earlier research (Chang et al., 2003). Both semantically prototypical *animate[goal]-inanimate[theme]* ordered DOD primes (e.g., *the girl brought the monkey a ball*) and non-prototypical *inanimate[goal]-animate[theme]* DOD primes (e.g., *the girl brought the zoo a monkey*) yielded an equal number of DOD targets (and the same for PD targets following PD primes). Thus, priming of DOD and PD structures was not dependent on the animacy-semantic role mappings they contained. We consequently agree with Bock and Loebell (1990) that structural priming is not fundamentally driven by semantics-syntax interactions in adults and further argue that the same applies for children.

There was a significant three-way interaction indicating that animacy could moderate the magnitude of structural priming in 3 year olds. Linguistic representations specifying animacy-semantic role mappings did appear to influence priming to some extent. *Post hoc* analyses revealed a marginal increase (after correcting for multiple comparisons) in PD priming where both the prime and target contained non-prototypical (AN theme & IN goal) animacy-semantic role mappings suggesting the potential for error-based learning effects to occur where surprising content reflects the animacy characteristics of the noun (see Chang et al., 2006). Based on prototypical usage of the PD structure, in the context of a transfer event, children may expect prepositions to be followed by an animate final noun (e.g., *the boy brought the ball to the tiger*). When this expectation was not met (e.g., *the girl brought the monkey to the zoo*), surprisal occurred, leading to enhanced priming effects. Since younger children use more PD than DOD sentences (Rowland et al., 2012), they may be less sensitive to animacy mappings in DOD as compared to PD sentences, and consequently have weaker expectations about the animacy characteristics of the nouns heard in DOD sentences, reducing the impact of semantics on priming.

We found no effect of animacy-semantic role mappings on the magnitude of priming in 5 year olds, contrary to Vasilyeva and Gámez (2015), but in line with our predictions about the likely developmental trajectory for semantic influences on priming. After testing a much larger sample size with prime-target match in animacy-semantic role mappings as a within-subjects variable, we suggest that animacy effects, at this later stage in development, might not be particularly reliable or generalizable to dative constructions. Our results do, however, provide support for the results of Huang et al. (2016) as we also found that animacy did not influence the magnitude of structural priming in adults, even after controlling animacy in themes and not just goals. We therefore suggest a developmental account whereby animacy effects on structural priming reduce with age. This fits with earlier findings that the effects of animacy-semantic role mappings on the accuracy of children's sentence interpretations decreased with age (Corrigan, 1988; Chan et al., 2009).

Nevertheless, animacy-syntax interactions did influence choice of dative constructions in 5 year olds and adults. They produced more DOD as opposed to PD targets where targets contained prototypical (AN goal & IN theme) mappings, regardless of prime structure, although 5 year olds only did so if the prime also contained prototypical mappings. This is consistent with the overall preference in adult speakers to use DOD rather than PD structures with dative verbs in conversational situations where prototypical mappings are expected to be frequent, and with children's apparently earlier mastery of the PD construction (e.g., Rowland and Noble, 2010; Rowland et al., 2012). Older participants are thus likely to be more sensitive to animacy mappings in DOD sentences, whereas younger children have yet to learn the precise semantic mappings for this construction (Rowland et al., 2012).

We show that the dative alternation is subject to animacy-semantic role effects but that beyond the earliest stages of language acquisition, animacy-semantic role mappings do not appear to influence the magnitude of structural priming. However, evidence suggests that animacy might exert an influence on sentence production independently of syntactic representations - speakers tend to alternate between different syntactic structures (e.g., active and passive) in order to maintain animate-inanimate noun ordering (e.g., adults: Gennari et al., 2012; children: Dewart, 1979). In Analysis two, we therefore assessed whether noun animacy orders can be primed independently of syntactic frames and without prime-target match in animacy-semantic role mappings in order to gain further insight into the effects of animacy on target word orders.

## Analysis 2: noun animacy priming

### Design

The study used a 3 × 2 × 2 × 2 mixed design. Age (3/5 years/adults) was a between-subject independent variable. Within-subjects variables were: prime animacy noun order (animate-inanimate [AN.IN]/inanimate-animate [IN.AN]), prime animacy-semantic role mappings (prototypical [AN goal − IN theme]/ non-prototypical [AN theme − IN goal] and prime-target match in animacy-semantic role mappings (match/mismatch). AN.IN noun ordering is used in prototypical DOD sentences (e.g., *the girl brought the monkey a ball*) and non-prototypical PD sentences (e.g., *the girl brought the monkey to the zoo*). IN.AN noun ordering is used in prototypical PD sentences (e.g., *the girl brought the ball to the monkey*) and non-prototypical DOD sentences (e.g., *the girl brought the zoo a monkey*). The proportional production of AN.IN responses was the dependent variable. See Table 6 for an overview of how prime and target sentences mapped onto the animacy variables for Analysis two.

**Table 6:** Examples of prime sentences and target elicitation scenes for each condition.

**Condition**	**AN.IN Prime noun order**	**IN.AN Prime Noun order**	**Target Elicitation Scene**
Prototypical Prime (AN goal & IN theme)/ Matched Target	*The girl brought the monkey a ball*	*The girl brought a ball to the monkey*	Transfer of a flower from a boy to a snail
Prototypical Prime (AN goal & IN theme)/ Mismatched Target	*The girl brought the bee a flower*	*The girl brought a flower to the bee*	Transfer of a monkey from a boy to a zoo
Non-prototypical Prime (IN goal & AN theme)/ Matched Target	*The girl brought a tiger to the zoo*	*The girl brought the zoo a tiger*	Transfer of a bee from a boy to a zoo
Non-prototypical Prime (IN goal & AN theme)/ Mismatched Target	*The girl brought a snail to the garden*	*The girl brought the garden a snail*	Transfer of a ball from a boy to a tiger

### Coding

Target responses were coded for animacy noun ordering independently of syntactic structure. As a result, responses that were excluded from Analysis one because they failed to adhere to either a PD or DOD structure were included in this analysis as long as they met the criteria listed below. Objects could be incorrectly named if this was due to a vocabulary error and not a misunderstanding of an objects' animate status or the event itself. Target responses that were initially prompted by the experimenter were included in the analyses. Sentences were coded as follows:

1. AN.IN sentences: contained animate before inanimate objects (e.g., the boy brought the tiger a ball/the boy showed the pig to a hutch//the boy threw the whale into the sea).
2. IN.AN sentences: contained inanimate before animate objects (e.g., the boy threw the food to the fish/the boy gave the zoo a tiger/the boy brought the ball next to the tiger).
3. OTHER: these were excluded from the analysis and included:
  - intransitives and/or incomplete sentences with only one noun (e.g., *the boy threw the whale*).
  - sentences where themes and goals did not differ in animacy (e.g., the boy threw the snack to the cactus/the boy gave the frog to the other frogs).
  - sentences in which the animacy status of at least one object was ambiguous (e.g., *the boy threw the thing to the sea*).
  - sentences where nouns were assigned to the wrong thematic roles since target animacy noun ordering may have been influenced by a misunderstanding of the target scene (e.g., *the boy brought the bee a zoo* after a target scene showing the transfer of a bee [theme] between a boy and a zoo [goal]).
  - sentences where nouns were incorrectly named due to participants' failure to note the animacy of objects or the nature of the event itself, rather than an error of vocabulary (e.g., *the boy brought the zoo/mouse a ball* instead of *the boy brought the tiger a ball*).

The percentage of OTHER target responses was 31% in the 3 year old group, 17% in 5 year olds and 9% in adults.

### Results

We examined whether animacy noun order could be primed independently of syntactic structure and assessed the extent to which the magnitude of this priming effect was dependent on whether (i) animacy-semantic role mappings are prototypical or non- prototypical and (ii) there is a match or mismatch in animacy-semantic role mappings across prime sentences and target scenes. We also examined whether there were any developmental changes in priming.

**Figure 4:**
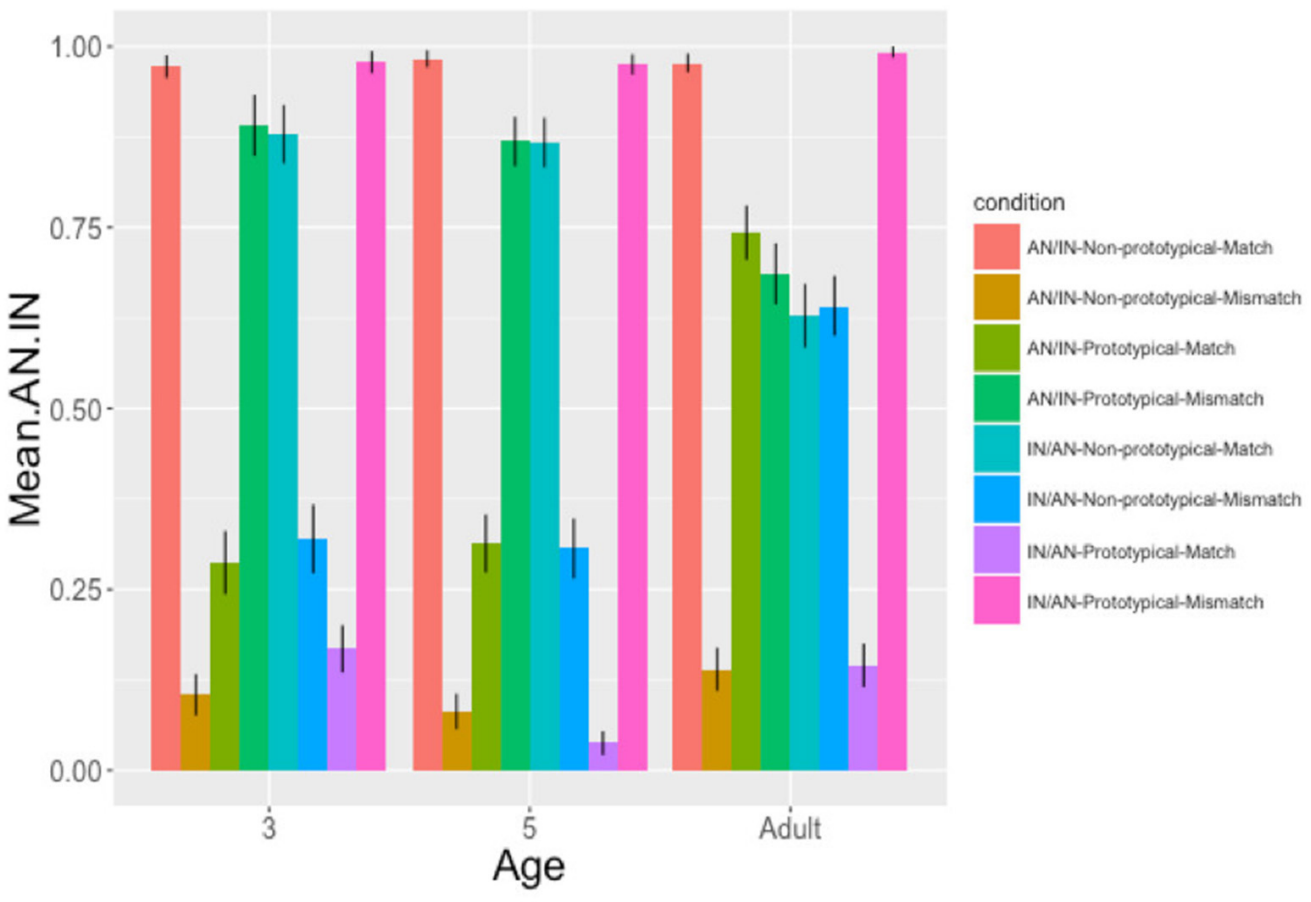
The mean proportion of AN.IN responses following AN.IN and IN.AN primes where primes contained either prototypical or non-prototypical animacy-semantic role mappings and these mappings were either matched or mismatched across primes and targets (SE in error bars).

The data were analyzed using logistic mixed effects models in *R*. The dependent variable was the production of target responses containing animate before inanimate objects ([AN.IN] = 1, [IN.AN] = 0). Fixed effects included: age (3 years = −1; 5 years = 0; adult = 1), prime animacy noun order (AN.IN = 1; IN.AN = 0), prime animacy-semantic role mappings (prototypical [AN theme − IN goal] = 1; non-prototypical [IN theme − AN goal] = 0) and prime-target match in animacy-semantic role mappings (match = 1; mismatch = 0). All variables were centered to reduce multicollinearity (Neter et al., 1985). Participant and item were included as random effects, but item was subsequently removed as the models failed to converge. Interaction terms were added to the models and retained if they improved model fit, as determined by ANOVA comparisons. The analyses were separated by age since the models initially fitted to the full data set including interactions with age did not converge. Within each age group, further models were run to explore each significant interaction effect using the Bonferroni correction and all *p*-values for individual predictors were obtained from the lme4 model summary output. Finally, where the results indicated a difference between children and adults, models comparing the 3 year olds and adults were run to confirm the presence of interactions between the manipulated sentence-level variables and age. The mean proportion of AN.IN target responses given in each experimental condition is shown in Figure 4.

#### Ages 3 and 5

Two separate models were run for children aged three and five. Both models initially contained only main effects of prime animacy noun order, prime animacy-semantic role mappings and prime-target match, but were significantly improved by adding two-way interaction terms between the variables (*p*s < 0.001). We found two significant two-way interactions and these were the same for both age groups. The first interaction was between prime animacy noun order and prime-target match; the second interaction was between prime animacy-semantic role mapping and prime-target match (see Table 7). All post hoc analyses were conducted using the Bonferroni method and a corrected alpha level of 0.025.

**Table 7:** Mixed effects logistic model results for children aged 3 and 5.

**Fixed Effects**	**Age 3**				**Age 5**			
	**Estimate**	**Standard Error**	***z*-value**	***p*-value**	**Estimate**	**Standard Error**	***z*-value**	***p*-value**
Intercept	0.78	0.16	4.79	0.001^**^	0.55	0.15	3.62	0.001^**^
Prime Animacy Noun order	−0.30	0.23	−1.34	0.181	0.37	0.27	1.38	0.167
Prime Animacy-Semantic Role Mapping	0.22	0.29	0.78	0.435	−0.33	0.28	−1.14	0.253
Prime-Target Match	−0.03	0.28	−0.12	0.903	−0.02	0.22	−0.13	0.9
Prime Animacy Noun order ^*^ Prime Animacy-Semantic Role Mapping	−0.65	0.64	−1.00	0.316	0.01	0.60	0.02	0.984
Prime Animacy Noun Order^*^ Prime-Target Match	2.78	0.61	4.57	0.001^**^	4.06	0.60	6.74	0.001^**^
Prime Animacy-Semantic Role Mapping ^*^ Prime Target Match	−8.71	0.64	−13.44	0.001^**^	−9.49	0.62	−15.28	0.001^**^

** *p < 0.001*.

To interpret the interactions between prime animacy noun order and prime-target match in animacy-semantic role mappings, a model was fitted for each level of animacy noun order (AN.IN and IN.AN) for each age group. For AN.IN primes there was a significant effect of prime-target match for both age groups (age 3: β = 1.11 (*SE* = 0.21), *z* = 5.21, *p* < 0.*0*01; age 5: β = 0.80 (*SE* = 0.19), *z* = 4.25, *p* < *0.0*01). AN.IN target production was greater where there was a match (age 3: *M* = 0.65, *SE* = 0.03; age 5: *M* = 0.64, *SE* = 0.03) as opposed to a mismatch in animacy-semantic role mappings (age 3: *M* = 0.37, *SE* = 0.04; age 5: *M* = 0.45, *SE* = 0.03). Both age groups also showed a significant main effect of prime-target match for IN.AN primes but in the opposite direction (age 3: β = −0.96 (*SE* = 0.21), *z* = −4.56, *p* < 0.001; age 5; β = −1.01 (*SE* = 0.19), *z* = −5.34, *p* < 0.001). More AN.IN targets were produced where there was a mismatch (age 3: *M* = 0.64, *SE* = 0.04; age 5: *M* = 0.64, *SE* = 0.03) rather than match (age 3: *M* = 0.42, *SE* = 0.04; age 5: *M* = 0.39, *SE* = 0.03) in prime-target animacy-semantic role mappings, i.e., children produced more IN.AN targets after IN.AN primes when the target matched the prime in its animacy-semantic role mappings.

To interpret the interaction between prime animacy-semantic role mappings and prime-target match, two models were fitted for each level of prime animacy-semantic role mapping (prototypical [AN goal & IN theme]/non-prototypical [AN theme & IN goal]) for each age group. For primes with prototypical animacy-semantic role mappings there was a significant effect of prime-target match, (age 3: β = −4.17 (*SE* = 0.43), *z* = −9.58, *p* < 0.*0*01; age 5: β = −4.21 (*SE* = 0.35), *z* = −11.91, *p* < 0.001). Fewer AN.IN targets were produced when target scenes contained matching (prototypical, age 3: *M* = 0.24, *SE* = 0.03; age 5: *M* = 0.18, *SE* = 0.02) as opposed to mismatching (non-prototypical, age 3: *M* = 0.95, *SE* = 0.02; age 5: *M* = 0.93, *SE* = 0.02) animacy-semantic role mappings. In both age groups, there was also a significant effect of prime-target match for primes containing non-prototypical animacy-semantic role mappings but in the opposite direction (age 3: β = 4.60, (*SE* = 0.48), *z* = 9.57, *p* < 0.001: age 5: β = 0.80 (*SE* = 0.19), *z* = 4.25, *p* < 0.001). More AN.IN targets were produced where target scenes contained matching (non-prototypical, age 3: *M* = 0.94, *SE* = 0.02; age 5: *M* = 0.94, *SE* = 0.02) as opposed to mismatching (prototypical, age 3: *M* = 0.20, *SE* = 0.03; age 5: *M* = 0.20, *SE* = 0.03) animacy-semantic role mappings.

To summarize, both 3 and 5 year olds produced identical results. They showed animacy noun priming, producing more AN.IN targets following AN.IN primes, and fewer AN.IN targets (i.e., more IN.AN targets) following IN.AN primes, but only where primes and targets contained matching animacy-semantic role mappings. In addition, 3 and 5 year olds produced more AN.IN targets after primes containing prototypical animacy-semantic role mappings (AN goal & IN theme) if the target contained non-prototypical (mismatching AN theme & IN goal) animacy-semantic role mappings compared to matching mappings. In contrast, for primes containing non-prototypical animacy-semantic role mappings (AN theme & IN goal), children produced more AN.IN targets if the target contained matching (non-prototypical) rather than mismatching (prototypical AN goal & IN theme) mappings.

#### Adults

Only main effects were included in the analyses since the model failed to converge with the addition of two-way interaction terms. The analyses revealed no significant effects as shown in Table 8. To summarise, animacy-semantic role mappings in primes and targets had no influence at all on animacy noun ordering in adults' target sentences.

**Table 8:** Mixed effects logistic model results for adults.

**Fixed Effects**	**Estimate**	**Standard Error**	***z*-value**	***p*-value**
Intercept	0.51	0.10	4.75	0.001^**^
Prime Animacy Argument Structure	0.21	0.13	1.60	0.11
Prime Animacy-Semantic Role Mapping	0.19	0.13	1.46	0.145
Prime-Target Match	0.05	0.13	0.42	0.676

** *p < 0.001*.

### Overall summary

Three and five year olds showed noun animacy order priming effects as they produced more *animate-inanimate* noun orders as compared with *inanimate-animate* noun orders following primes with *animate-inanimate* noun orders. However, this only occurred where there was prime-target match in animacy-semantic role mappings. The magnitude of priming was not influenced by whether mappings were prototypical (AN goal & IN theme) or non-prototypical (AN theme & IN goal). Independently of noun animacy order priming, both groups of children produced fewer *animate-inanimate* (more *inanimate-animate*) sentences where primes and targets contained matching prototypical (AN goal & IN theme) mappings and more *animate-inanimate* sentences where mappings in primes and targets were matched and non-prototypical (AN theme & IN goal). Adults showed no noun animacy order priming effects, and animacy-semantic role mappings had no influence on target animacy noun orders. Examination of Figure 4 suggests that the largest difference between the children and adults lies in their responses to AN.IN-prototypical primes (prime-target match vs. mismatch) and IN.AN non-prototypical primes (prime-target match vs. mismatch). To confirm that there were developmental differences, two models were run to test for an interaction between age and prime-target match for these prime types. The models revealed significant interactions (AN.IN-prototypical age^*^match β = 3.31, *p* < 0.001; IN.AN-non-prototypical age^*^match β = −2.86, *p* < 0.001). While the adults were equally likely to produce AN.IN targets following all prime types, the 3 year olds produced more AN.IN responses for targets containing animate themes and inanimate goals than for targets containing inanimate themes and animate goals, demonstrate a strong tendency to place themes before goals irrespective of the animacy-semantic role mappings of the prime.

### Interim discussion

This study provides evidence of animacy noun priming effects in 3 and 5 year olds. We show that children can be primed to reuse noun animacy orders in target sentences regardless of whether or not they repeat prime syntactic structures. However, our results indicate that animacy cues do not work independently of semantic roles to achieve priming effects. If animacy cues worked alone, children would be primed to produce AN.IN targets following AN.IN primes, irrespective of prime-target match or mismatch in animacy-semantic role mappings. Instead, animacy noun priming was driven by representations specifying animacy-semantic role mappings (e.g., *animate goal-inanimate theme* as opposed to merely *animate-inanimate*) and occurred as a result of the reuse of these mappings in the specified order. However, priming was not enhanced by error-based learning mechanisms or dependent on the use of prototypical mappings since the magnitude of noun animacy priming did not differ depending on whether animacy-semantic role mappings were prototypical or non-prototypical.

Furthermore, our study reveals that children construct sentences according to a preferred semantic role structure. Their preference to use *theme-goal*, as opposed to *goal-theme* structures overrides any preference for a particular noun animacy order. Where there was prime-target match in prototypical (AN goal & IN theme) animacy-semantic role mappings, 3 and 5 year olds tended to produce IN.AN targets (e.g., *the boy brought the flower* [theme] *to the snail* [goal]). Where there was prime-target match in non-prototypical (AN theme & IN goal) mappings, both groups of children tended to construct AN.IN targets (e.g., *the boy brought the cow* [theme] *to the farm* [goal]). This is consistent with the claim that children use *theme-goal* ordered PD constructions more frequently than *goal-theme* ordered DOD constructions (Rowland et al., 2012).

This finding is however, at odds with Bock and Warren's (1985) conceptual accessibility theory which predicts a general preference for AN.IN sentences that might be expected to override a preference for a particular semantic role structure. Representations of animate objects are thought to be retrieved and processed more quickly than representations of inanimate objects during sentence formation such that animates are ordered before inanimates. There is supporting evidence from research on the active/passive alternation in children (Dewart, 1979) and adults (Gennari et al., 2012) but our results suggest that conceptual accessibility effects might not extend fully to sentences with two post-verbal nouns, even in young children.

Noun animacy order effects were subject to a developmental decrease to the extent that adults showed no evidence of priming. This fits in with claims that speakers' sensitivity to semantic content in sentences decreases with age (see Corrigan, 1988; Savage et al., 2003). Although our results contrast with those of Bock et al. (1992) who found priming effects of noun animacy order in adults, we suggest that this is because animacy noun priming might be moderated by syntactic structure. We used dative sentences while Bock et al. (1992) used actives and passives. Where target sentences contain one pre-verbal and one post-verbal noun, as opposed to two post-verbal nouns, animacy cues might exert stronger effects on target animacy noun orders.

## General discussion

### Full results summary

In this paper, we investigated the extent to which semantic information in the form of thematic role and animacy characteristics influenced sentence processing through interactions with, and independently of, syntax. The goal was to cast light on the nature of children's and adults' sentence representations, and to determine whether these change developmentally. Whilst theories of adult sentence processing offer conflicting accounts of how and when semantic and syntactic information influence sentence production (independently, e.g., Pickering and Branigan, 1998; Kaschak and Glenberg, 2004, or in interaction, e.g., Hare and Goldberg, 1999; Chang et al., 2003), there is reason to believe that children's sentence representations might be more closely tied to the semantic characteristics of the sentences they encounter most often (e.g., Ambridge et al., 2015).

In Analysis one, we assessed whether the magnitude of structural priming varied depending on whether animacy-semantic role mappings were prototypical (AN goal & IN theme) or non-prototypical (AN theme & IN goal) and matched or mismatched across primes and targets. We also investigated whether priming effects are subject to developmental change. Our results showed structural priming effects in all age groups, as well as evidence of a marginally enhanced priming effect for PD primes (but not DOD primes) in our 3 year olds where primes and targets contained matching, non-prototypical (AN theme & IN goal) animacy-semantic role mappings. Animacy-semantic role mappings did not affect structural priming in 5 year olds and adults. Nevertheless, 5 year olds and adults produced significantly more DOD as opposed to PD target sentences where the target contained prototypical animacy semantic role mappings (AN goal & IN theme), although in the case of 5 year olds, this only occurred where there were matching prototypical primes.

In Analysis two, we examined whether participants could be primed to reuse animacy noun orders in their target sentences independently of syntactic structure. We also investigated whether the magnitude of animacy noun priming was influenced by whether animacy semantic role mappings were prototypical (AN goal & IN theme) or non-prototypical (AN theme & IN goal) and matched or mismatched across primes and targets. Furthermore, we assessed whether priming effects changed over the course of development. Our results showed significant noun animacy priming effects in 3 and 5 year olds, where there was prime-target match in animacy-semantic role mappings. It did not matter whether mappings were prototypical (AN goal & IN theme) or non-prototypical (AN theme & IN goal). The children produced fewer AN.IN (more IN.AN) target sentences where there was prime-target match in prototypical (AN goal & IN theme) mappings, but more AN.IN (fewer IN.AN) sentences where there was prime-target match in non-prototypical mappings (AN theme & IN goal). Together these results indicate a preference to construct sentences with themes before goals. Adults, however, showed no animacy noun priming effects and animacy-semantic role mappings had no influence on target animacy noun orders.

### Theoretical implications

Our structural priming results largely support accounts that posit independent effects of syntax and semantics in priming. However, there is evidence that the role of semantic information in priming may change over development. We found a significant three-way interaction in our 3 year olds (Analysis one) between syntactic structure, animacy-semantic role mappings, and prime-target match. Although post-hoc analyses corrected for multiple comparisons revealed only a marginal effect of semantic information on the priming of PD (but not DOD) structures, the overall pattern in the data suggests that these young children have a stronger grasp of the PD structure than the DOD structure, in line with previous research (e.g., Rowland et al., 2012). On the basis of these data, we should be cautious in what conclusions we draw from the apparent interaction between semantic and syntactic information. However, if this finding can be replicated, this would provide evidence which is at odds with theories of structural priming which argue for an autonomous syntax, at least during the early stages of language acquisition. Activation models (Pickering and Branigan, 1998; Kaschak and Glenberg, 2004) do not consider the possibility that linguistic representations might specify animacy cues that could facilitate structural priming. Instead, they attribute structural priming effects to a reactivation of combinatorial nodes and connected lemma nodes representing dative verbs that were used in prime sentences (Pickering and Branigan, 1998) or to the easier retrieval from memory of recently activated prime structures (Kaschak and Glenberg, 2004). Our data are more consistent with models which allow for some degree of interaction between semantic and syntactic processes in sentence production such as implicit learning and adaptation models (e.g., Chang et al., 2006; Jaeger and Snider, 2013). Thus, animacy-syntax interactions might not fundamentally drive priming, since there was a main effect of prime structure in all age groups but our evidence suggests that they do have the potential to facilitate priming in 3 year olds, consistent with usage-based approaches.

We also found evidence for the independent effects of semantic information on priming, irrespective of syntactic structure. Our 5 year olds and adults both showed stronger priming effects (a preference to use DOD structures) when targets contained prototypical as opposed to non-prototypical animacy-semantic role mappings. However, for the 5 year olds, this effect was only observed when the prime sentence also contained prototypical semantic mappings. Here, then, we observe further evidence for a developmental change in semantics-syntax associations. Only our 5 year old children have begun to associate prototypical animacy-semantic role mappings (AN goal, IN theme) with the DOD structure, but unlike adults, this association is dependent on having recently heard a sentence modeling prototypical semantic mappings. Taken together, the data from Analysis one suggest that there are developmental changes in the strength of the associative links between the semantic characteristics of events and the sentence structures used to describe them which warrant further investigation.

Our Analysis one data also speak to the question of whether error-based learning contributes to larger priming effects (Chang et al., 2006). This model predicts greater priming effects to occur after primes containing unexpected (and hence more salient and memorable) content. Although there is some evidence for enhanced priming effects following unexpected syntactic structures (e.g., DOD primes for verbs biased toward the PD structure, Peter et al., 2015), it is unclear whether similar effects might occur at the level of unexpected animacy-semantic role mappings. Our finding that PD sentence priming in 3 year olds was marginally increased with non-prototypical (AN theme & IN goal) animacy-semantic role mappings where there was prime-target match indicates the potential for error-based learning to occur where animacy-semantic role mappings constitute the surprising content.

Our investigation of whether there are semantic influences on priming in the form of noun animacy ordering, independent of syntactic structure (Analysis two), reveals a further interesting developmental trajectory. We found that noun animacy order priming occurred in children, but not in adults, for both prototypical and non-prototypical animacy-semantic role mappings although effects were only observed in the children where there was prime-target match. In addition, for non-prototypical targets, the children preferred to place animate themes before inanimate goals, irrespective of whether this matched or mismatched the mappings in the prime. Taken together, these data suggest that noun animacy cues do not act independently of semantic roles (themes & goals), even at the early stages of language acquisition, but rather interact with semantic role-grammatical function mappings (de Swart et al., 2008). In line with our predictions derived from usage-based approaches, these syntax-independent noun animacy priming effects are no longer evident in adulthood, at least using a sentence production methodology.

### Future research

We provide evidence to suggest that animacy cues are specified in children's linguistic representations in such a way as to influence word orders in the sentences that they produce. However, in order to gain a deeper understanding of animacy effects on sentence processing, researchers must also seek to identify how fine-grained animacy noun specifications might be and whether they can influence sentence comprehension. Chan et al. (2009) reported that 2 year olds interpret active sentences with animate agents and inanimate patients more accurately than sentences with inanimate agents and animate patients. It thus seems that children's representations of agent roles were more likely to specify animate nouns than inanimate nouns while patient roles tended to specify inanimate nouns. Nevertheless, it is unclear how fine-grained these animacy specifications are since researchers have often assumed a binary distinction between animates and inanimates. In contrast, Demuth et al. (2005) provide evidence to suggest that children's syntactic categories specify noun animacy according to a fine-grained animacy hierarchy. They found that children's comprehension of sentences in Sesotho was guided by animacy hierarchy effects where nouns are ranked as follows: human>animal>inanimate (see Comrie, 1989). Children were better able to comprehend sentences with human benefactives and inanimate themes than sentences with animal benefactives and inanimate themes. Further experimental research is required in order to clarify whether animacy hierarchy effects influence English sentence comprehension, and how these properties might impact on sentence representations and consequently on priming effects.

## Conclusion

Our study provides important new evidence demonstrating how structural priming works. While structural priming appears fundamentally dependent on the repetition of syntactic frames and not the order of animacy-semantic role mappings, animacy-syntax interactions moderated the magnitude of priming in young children. Furthermore, although animacy effects on structural priming were subject to a developmental decrease, the extent to which animacy-semantic role mappings influenced speakers' choice of dative structure independent of structural priming, and independent of a match in semantic role mappings between primes and targets increased with age into adulthood. Animacy noun orders could also be primed in children but not adults, but these effects were tied to aspects of the semantic role mappings involved. Animacy cues can hence affect speakers' word orders independently of syntactic structures and perhaps also through interactions with syntax, although these processes are subject to developmental changes. We therefore, suggest that theories of structural priming, sentence production, linguistic representation and language acquisition all need to explicitly account for developmental changes in the role of semantic and syntactic information in sentence processing.

## Ethics statement

This study was carried out in accordance with the recommendations of the British Psychological Society. Written informed consent from the Head teachers of early years educational settings and/or parent caregivers of the children concerned was obtained along with written consent for adult participants, and verbal assent for child participants. The protocol was approved by The University of Manchester Research Ethics Committee (UREC, project no. 15255).

## Author contributions

All authors made substantial contributions to the conception and design of the work, the drafting and/or revising of the work, and have approved the final manuscript. LB carried out data collection and analysis.

### Conflict of interest statement

The authors declare that the research was conducted in the absence of any commercial or financial relationships that could be construed as a potential conflict of interest.
